# The impact of an exercise and sport intervention on cognitive function and pain among forcibly displaced individuals at risk for PTSD: a secondary analysis of the SALEEM randomized controlled trial

**DOI:** 10.1186/s12916-024-03601-x

**Published:** 2024-09-12

**Authors:** Florian Knappe, Konstantinia Filippou, Antonis Hatzigeorgiadis, Ioannis D. Morres, Sebastian Ludyga, Harald Seelig, Emmanouil Tzormpatzakis, Elsa Havas, Yannis Theodorakis, Roland von Känel, Uwe Pühse, Markus Gerber

**Affiliations:** 1 Department of Sport, Exercise and Health, University of Basel, Grosse Alle 6, 4052 Basel, Switzerland; 2 Department of Physical Education and Sport Sciences, University of Thessaly, 42100 Trikala, Greece; 3 Department of Nutrition and Dietetics, University of Thessaly, 42132 Trikala, Greece; 4 Department of Consultation-Liaison Psychiatry and Psychosomatic Medicine, University Hospital Zurich, University of Zurich, 8091 Zurich, Switzerland

**Keywords:** Executive function, Fitness, Migrant, Physical activity, Public health, Refugee camp

## Abstract

**Background:**

In response to the global scope of forced displacement, international organizations highlight the need of scalable solutions to support individuals’ health and integration into host societies. Exposure to high mental and physical stress perceived before, during, and after displacement can impair functional capabilities, essential for adapting to a new environment. This secondary analysis examined the impact of an exercise and sport intervention on cognitive function and pain severity among individuals living in a refugee camp in Greece.

**Methods:**

We implemented a randomized controlled trial involving *n* = 142 (52.8% women) forcibly displaced individuals from Southwest Asia and Sub-Saharan Africa. Participants were randomly assigned to a waitlist or a 10-week co-designed exercise and sport intervention with a 1:1 allocation rate between groups and sexes. Assessments at baseline and follow-up included the Flanker task, the Oddball paradigm, pain severity via visual analog scales, and the Åstrand-Rhyming indirect test of maximal oxygen uptake. We analyzed the intervention effects using structural equation modeling.

**Results:**

Our findings did not indicate a direct intervention effect on cognitive function or pain (*p* ≥ .332). However, the intervention group significantly improved cardiorespiratory fitness, *ß* = .17, *p* = .010, which was associated with faster reaction times in cognitive tasks, *ß* =  − .22, *p* = .004. Moreover, there was some evidence that adherence might be linked to reduced pain severity, *ß* =  − .14, *p* = .065.

**Conclusions:**

Exercise and sport did not directly impact cognitive function and pain severity among a sociodemographically diverse sample living in a refugee camp, suggesting the need for complementary measures. Nevertheless, our results indicate that improvements in cardiorespiratory fitness benefit aspects of attention.

**Trial registration:**

The study was approved by the local ethics committee of the University of Thessaly (no. 39) and registered prospectively on February 8, 2021 at the ISRCTN registry (no. 16291983).

**Supplementary Information:**

The online version contains supplementary material available at 10.1186/s12916-024-03601-x.

## Background

Based on UNHCR’s report [1], the global rate of forced displacement increased, with an average of 36.3 individuals per minute leaving their homes due to global political, economic, and environmental factors. This led to worldwide 108.4 million forcibly displaced individuals in 2022, which is more than a two-and-a-half-fold rise over the past decade. Nearly 90% of displaced individuals reside in low- and middle-income countries, where displacement predominantly occurs. Besides that, there is an observable increase in the proportion of forcibly displaced individuals in European countries [2]. Due to its geographic location near Asia, Greece is an important entry point.

Individuals forced to leave their countries positively impact the economies of European host countries [3]. This is opposite to recent perceptions that forcibly displaced individuals are an economic burden and exploit local welfare services [4]. Instead, they bridge critical labor gaps in high- and low-skilled sectors, notably health, care, and cleaning services [2].

However, integrating into a new society poses significant challenges for individuals granted asylum, particularly in mastering the local language and achieving financial independence [5]. Yet, the high functional requirements for social integration are often challenged by exposure to high levels of mental and physical stress experienced before, during, and after displacement. Experiences can range from exposure to traumatic events like war and torture to challenges faced during the journey, including physical or sexual abuse, harsh detention conditions, legal uncertainties, work restrictions, or discrimination [6]. These stressors have a profound impact on mental and physical health, evidenced by a markedly higher prevalence of mental disorders, particularly post-traumatic stress disorder (PTSD), among forcibly displaced populations compared to non-displaced groups [7, 8].

Recent studies suggest a connection between severe PTSD symptoms and cognitive difficulties among forcibly displaced individuals, including declines in sustained attention and inhibitory control [9, 10]. Sustained attention, the ability to continuously focus on specific stimuli, is believed to be fundamental for gains in executive function [11]. Inhibitory control is a component of executive function that enables one to selectively focus and resist distractions and thus actively maintain a task or reach a goal [12]. Executive functions are associated with quality of life, academic achievements, and employment stability [13], essential for adapting to new environments. Among trauma-affected individuals, inhibitory control gains increased relevance, given that intrusion and hyperarousal are central components of PTSD [14]. Observations suggest a link between improved inhibitory control and reduced PTSD symptoms, yet the nature of this relationship remains unclear [14].

In addition to the increased mental burden, chronic pain is more than twice as prevalent among forcibly displaced individuals (43%) compared to the broader population (20%), which further complicates the situation of the former [15, 16]. Chronic pain cannot only lead to functional impairment, but is also linked to increased PTSD symptoms [17]. This relationship, potentially bidirectional and influenced by a set of psychological, biological, behavioral, and cultural factors, suggests that alleviating pain is crucial in treating PTSD effectively [17–19].

Considering their intertwined relationship, addressing cognitive function and chronic pain may not only improve PTSD treatment outcomes, but also serve as protective factors against functional impairments amid the challenges of post-migration living difficulties. Therefore, early measures could contribute to an individual’s right to health [20] and foster socio-economic integration, allowing individuals to actively contribute to their host society [21]. Nevertheless, affected individuals still face a shortfall in receiving sufficient healthcare support [22].

The WHO advocates for evidence-based measures to meet the health needs of forcibly displaced individuals. In their latest research agenda on health, migration, and displacement [23], they stress the importance of multisectoral approaches to improve health care provision in detention centers and camps. The global scope of forced displacement requires effective and scalable solutions [22]. Exercise and sport appear as feasible add-ons, known for their broad health benefits [24], including improvements in cognitive function and pain reduction [16, 25]. Higher fitness levels are linked to improved cognitive function through heightened cerebral blood flow, neuroplasticity, and increased gray and white matter volume [13, 26]. Additionally, in individuals experiencing chronic pain, cardiorespiratory fitness (CRF) is associated with reduced pain severity, although this relationship is to date disputed [27].

To our knowledge, no study has examined so far how exercise and sport impact cognitive function in individuals living in refugee camps. Furthermore, only two studies have assessed the effect of exercise on pain among forcibly displaced individuals, as a supplementary treatment to cognitive behavioral therapy in clinical settings [28, 29]. Considering the unique challenges of post-migration living difficulties and restrained resources in refugee camps, examining exercise and sport as a potential first-line approach in this setting seems needed.

This study aims to evaluate the effects of a co-designed [30] exercise and sport intervention on cognitive function and pain among forcibly displaced individuals living in a refugee camp in Greece. Additionally, we explore the potential risk of adverse events related to the intervention. Reflecting observations in the broader population, the effectiveness of these interventions could further be compromised by low exercise adherence among forcibly displaced individuals [31]. We expect that the intervention will lead to improvements in cognitive function and pain, primarily driven by enhancements in CRF and adherence with exercise.

## Methods

### Study design

This study examined a subset of data from a larger pragmatic randomized controlled trial in a refugee camp in Greece (ISRCTN16291983), which consisted of two parallel groups: an intervention group (IG) and a waitlist control group (WLCG). In addition to the primary outcome, which demonstrated a reduction in PTSD symptom severity linked to regular attendance over a 10-week intervention span [32], this paper presents the intervention effects on cognitive function and pain. The sampling and procedure of this study were consistent with the protocol in the registered project [33]. The documentation of our study adhered to the Consolidated Standards of Reporting Trials (Additional file 1: Table S1). The University of Thessaly’s Research Ethics Committee provided ethical approval (no. 39).

### Setting

The study took place in a refugee camp located on mainland Greece, managed by the Ministry of Migration and Asylum. This camp served as a provisional residence for individuals awaiting the outcome of their asylum applications. Accommodation was provided in container units, which we refer to as “households.” The households were either occupied by families or up to four individuals of the same sex and origin. Healthcare services were provided by a healthcare facility within the camp staffed with two physicians, several nurses, and two psychologists.

As of February 2021, the camp’s population was recorded at 1376 individuals by the site management. Among them, 920 residents (67%) were aged between 16 and 59 years, with women comprising 39%. The camp’s inhabitants represented a wide array of sociodemographic backgrounds. The largest groups were from Afghanistan (45%) and Syria (25%), while 30% came from regions including West Asia (11%), Sub-Saharan Africa (17%), and other areas (2%).

### Participants

The study included persons who (a) resided in the specified refugee camp, (b) were aged between 16 and 59 years, (c) were able to read or communicate in English, Arabic, Farsi, or French, and (d) gave their written informed consent. In line with the trial’s pragmatic nature and adhering to ethical standards, the inclusion criteria were set comprehensively to minimize exclusion of the exercise and sport activities. The choice of languages (Arabic, Farsi, and French) reflected the predominant linguistic backgrounds of the camp residents. Sample size calculation was based on identifying a small effect of the intervention on PTSD symptoms, which was the primary outcome of the overarching study. It was estimated that at least 136 participants were needed, considering an anticipated dropout rate of 25% [33].

### Procedure

The study commenced in May 2021, encompassing initial screening, participant recruitment, baseline assessment (T1, week 0), and random allocation. The recruiting and allocation process was carried out by households to avoid the division of persons within the same household. Aiming to include as many households as possible, we conducted an extensive screening to capture the sociodemographic characteristics of the camp population. From the pool of eligible households, a sex-stratified sample was drawn. Additional households were drawn to compensate for potential non-attendance and achieve our calculated sample size. After T1, the Greek project coordinator randomly assigned the households to two groups, with a 1:1 allocation rate between groups and sexes employing a random number generator. Groups were then randomly designated as either part of the intervention or placed on a waitlist 3 days before the intervention started and notification of the participants. Screening, sampling, and allocation were assigned to different team members. This approach was employed to reduce the risk of selection bias.

Before data assessment, we sought written informed consent from potential study participants. They were given written and oral explanations about the study’s objectives and procedures. To support understanding, we presented a self-directed film illustrating the individual measurements involved in the assessments. It was emphasized that their involvement was entirely voluntary and that withdrawal would have no adverse consequences, particularly regarding their asylum application. Cultural and linguistic barriers resulting in potential misunderstandings and the creation of false expectations, however, could compromise the voluntary nature of participation [34]. To address these challenges and reduce associated risks, we recruited 10 female and male community translators within the camp population. Their role involved explaining the study procedures, ensuring informed consent, and providing translation services.

T1 and follow-up assessments (T2, week 11) were conducted at the Department of Physical Education and Sport Science at the University of Thessaly. After completing the assessment, participants received a briefing on their results. Additionally, as a form of compensation and to ensure reciprocal benefits from the research, participants were offered a meal and sports equipment, valued at approximately 40 euros.

### Intervention

Table 1 provides an overview of the intervention. The intervention was rooted in co-design and continuously tailored to suit individual, cultural, and situational conditions. Target group and stakeholder involvement is both ethically and practically vital. This approach not only prevents paternalism but also supports cultural and contextual suitability [35]. Furthermore, tailored interventions are increasingly acknowledged for effectively enhancing exercise participation and greater benefits for executive function and pain [13, 36].

**Table 1:** Overview of the intervention

Component	Detail	
Objective	Design and implement engaging and appealing exercise and sport activities that foster enjoyable experiences while supporting participant autonomy	
Intervention type	Provision of recreational exercise and sport activities based on the self-determination theory^a^ and co-developed^a^ with participants	
Frequency	1–2 activities per day, 5 days a week	
Duration	10 weeks, 50–60 min per session	
Intensity	Individually regulated based on preferences and capacities	
Activities	Typical activities for women per week	
	2 × fitness training	
	1 × martial arts	
	3 × ball sports (soccer, volleyball)	
	2 × dance	
	Typical activities for men per week	
	1 × fitness training	
	2 × martial arts	
	5 × ball sports (soccer, volleyball, basketball)	
Content	Warm-up (10–15 min)	
	Light jogging, small games, muscle activation exercises, dynamic stretching	
	Main activity (30–40 min)	
	Fitness training	Circuit training, group workouts, exercise games
	Martial arts	Technique training, training of martial arts-specific strength endurance, sparring
	Ball sports	Technique drills, tactical game-based drills, full game play
	Dance	Technique and choreography training, peer coaching of cultural dances
	Cool-down (5–10 min)	
	Easy movement, static stretching, breathing exercises	
Supervision	Each session guided by two postgraduate students in physical education of the same sex as participants, with support from community translators	

^a^According to the literature [30, 37]

Co-design was conceptualized as a collaborative process involving service users, staff, and camp management to enhance quality [30]. We utilized qualitative screening tools and engaged in numerous informal conversations with participants and stakeholders before starting the intervention. During the intervention, additional debriefing meetings were held between the coaches and project coordinators, discussing implementation challenges and potential solutions. This measure allowed timely adjustments to emerging difficulties. The intervention’s design and subsequent modifications covered aspects such as the type of activities, session content, the timing and location of sessions, and adjustments for changing climatic and structural conditions. Additional, measures were taken to overcome participation barriers and ensure accessibility, including organizing childcare services during activities or providing private spaces for women’s sessions.

The intervention provided a variety of activities held separately for women and men. Participants were free to choose their preferred activities and encouraged to engage in them regularly through personal interactions. Regular updates on upcoming activities were communicated through an internal chat group. In line with the self-determination theory [37], participants were encouraged to participate at their own pace in the activities, acknowledging individual preferences and capacities.

The activities took place inside and around the camp, utilizing existing infrastructure such as a soccer field, volleyball court, gymnastics room, or an empty storage hall. Given the local climatic conditions, the activities were scheduled during the cooler evening hours before Maghrib. Assuming a positive impact of the exercise and sport activities, we extended the intervention beyond the official trial period. Consequently, the activities were made available to the waitlist control group and all camp residents after the study.

### Measures

Adhering to a standard operating procedure, trained research staff assessed outcome variables, including cognitive function, pain, CRF, and PTSD symptom severity. Given the nature of the intervention and the dual roles of some assessors in implementing activities and collecting data, blinding to group assignments was impossible. Communication was facilitated by translators and questionnaires were provided in English, Arabic, Farsi, and French to accommodate the native language backgrounds of the participants.

#### Cognitive function

The assessment of sustained attention and inhibitory control was conducted with the Oddball paradigm [38] and the Flanker task [39], respectively. These cognitive assessments were performed using E-Prime 3.0 (PST, Pittsburgh, USA), a computer-based test and have been reported as reliable tools in neuropsychology [40, 41] for measuring cognitive function [42].

The tests were carried out in a separate room to ensure an environment that facilitates concentration, reducing surrounding noise and distraction. The testing sequence lasted between 15 and 20 min and was identical across all participants, involving the Oddball paradigm followed by the Flanker task. Each task began with a practice block to familiarize participants with the task, followed by two test blocks of 40 trials each, interrupted by a 30-s break. Data on reaction time and accuracy (for correct responses) were separately extracted for standard and rare trials on the Oddball task and congruent and incongruent trials on the Flanker task. Trials with response times at or below 120 ms were excluded from the calculation of the average due to the high risk of guesses. Preliminary analyses indicated marginal differences in effect size between the Oddball paradigm and the Flanker task (Additional file 1: Table S2). An overarching *Z*-score for reaction time and one for accuracy was then built by standardizing individual scores and computing the mean across both tasks.

In the Oddball paradigm, participants responded to standard (70%) and rare (30%) target stimuli by pressing a blue or yellow button, respectively. The visual stimuli “X” and “O” were displayed for 300 ms, with a variable inter-trial interval of 600 to 900 ms. Responses were recorded within 1050 ms after the stimulus appeared.

The Flanker task required participants to identify the direction of a central horizontal arrow (left or right) and to disregard the 4 flanking arrows, which either pointed in the same direction (congruent trials) or the opposite direction (incongruent trials). Both types of trials were presented with equal probability and in a random sequence. Each visual stimulus was shown for 300 ms, with an inter-trial interval varying between 900 and 1200 ms, and responses were collected within 1300 ms.

Originally, the 2-back test was intended for assessing working memory [33]. However, initial assessments indicated consistent inaccuracies in its execution. The 2-back test was subsequently removed from the test battery to reduce the participant’s test load. As this test was the last in the sequence, its removal did not affect performance in the preceding tests.

#### Pain

Pain severity was measured using the 5-item visual analog scale for pain (VAS) [43]. The VAS consists of a 100 mm horizontal line labeled at the ends with “no pain” (0 mm) and “severe pain” (100 mm). Participants reported their average pain experienced over the past week across different body regions, such as head, back, chest, stomach, and extremities, which have been the most prevalent chronic pain locations among a sample of forcibly displaced individuals [44]. A composite index was then built by computing the mean of these 5 measurements. Scores greater than 30 and 60 were considered moderate and severe pain, respectively [16]. This instrument has been validated in previous research [45] and used with forcibly displaced individuals [46].

#### Cardiorespiratory fitness

The Åstrand-Rhyming indirect test of maximal oxygen uptake was performed on a bicycle ergometer to assess CRF [47]. We included participant’s sex, age, body weight, mean steady state, and power output in calculating peak VO_2_max (ml/kg/min) [48]. The validity of the Åstrand-Rhyming test for estimating VO_2_max has been reported in earlier studies [49], and its protocol has been successfully applied with forcibly displaced individuals [50].

#### Symptoms of post-traumatic stress disorder

PTSD symptoms were assessed using the 22-item Impact of Event Scale-Revised (IES-R) [51], which aligns with the DSM-5 [52] and ICD-10 [53] criteria for PTSD. Respondents rated items on a 5-point Likert scale ranging from 0 (not at all) to 4 (extremely), resulting in a total score of 0 to 88. The IES-R was previously utilized among forcibly displaced individuals [54] and is valid and reliable in Arabic, English, Farsi, and French [51, 55–57].

#### Sociodemographic background

Participants self-reported sociodemographic characteristics, including sex, age, origin, educational background, and length of stay in the camp.

#### Adherence

Throughout the intervention, coaches recorded attendance at each session, tracking each participant’s total number and type of activities attended per week and during the study.

#### Adverse events

The importance of reporting adverse events in exercise research has recently been emphasized [58]. Coaches actively monitored any adverse outcomes occurring during activities, documenting them in the attendance records. Serious adverse events were classified as any incident resulting in death, hospitalization, or significant health deterioration. All other incidents were classified as non-serious [58]. In the event of adverse outcomes, coaches provided first aid and, if necessary, referred participants to the camp’s internal healthcare facility. Based on the event’s severity, precautions were taken, including adjustments of exercise parameters, a temporary pause, or the individual’s exclusion from the intervention.

### Statistical analysis

We utilized SPSS (version 29.0, IBM, Armonk, USA) and the AMOS interface for our analyses, encompassing both descriptive and inferential statistics. Data analysts were aware of the participant’s group allocation due to the included attendance records in the dataset. Initially, we screened data for uni- and multivariate outliers, using the Mahalanobis distance for the latter [59]. Normality was assessed through Kolmogorov–Smirnov tests, and log transformation was applied to non-normally distributed variables with skewness/kurtosis *z* values exceeding 3.29 [60].

Descriptive data is presented as mean (M), confidence intervals (95% CI), frequency (*n*), and relative proportions (%). We employed chi-square and independent samples *t*-tests to compare participants who completed the study at T2 with those who dropped out, examining differences in sociodemographic characteristics and outcome variables at T1. We further used Little’s missing completely at random test to examine missing data and performed additional analyses on missing data mechanisms to determine whether missing data at T2 depended on covariates or T1 values [61]. If Little’s test indicated that data were not missing completely at random, we compared groups with complete and incomplete datasets regarding covariates and outcome variables and incorporated significant covariates in our model [62]. In the case of ignorable missing data, the full information maximum likelihood approach was employed. This method was chosen due to its lower bias than other common methods [63].

Given the expected dropout due to the camp setting’s unique challenges [2], where participants may be forced or choose to leave the camp, we performed a completer analysis. This decision was based on the assumption that follow-up loss is random and unrelated to the treatment or studied condition [64]. We incorporated nonadherent participants further to mitigate post-randomization confounding and selection bias in our results. In contrast to our pilot study [65], the dropout rate in the current study was notably higher. Consequently, we decided not to proceed with the planned further analysis of the second (T3, week 21) and third (T4, week 31) follow-up assessments.

Intervention effects on cognitive function and pain were examined via post-scores, using a causal model and controlling for autoregressive effects. We applied structural equation modeling to examine the mechanisms underlying the intervention’s impact and to analyze multiple relationships within one model. Structural equation modeling differs from regression models by enabling variables to serve in dual roles as outcome and predictor variables in different model section, thereby reflecting causal pathways more accurately [63]. We modeled CRF as both an outcome of the intervention and a predictor of cognitive function and pain (model 1). An as-treated analysis was conducted to account for adherence, comparing individuals who participated in the activities once or more per week with those who did not participate instead of the assigned treatment groups (model 2).

Sex and age were included as covariates. Given the assumed association of PTSD with cognitive function and pain, we further controlled for PTSD symptom severity [9, 10, 14, 17–19]. Following model fit indices, outcomes were adjusted for covariates at T1 across all analyses. We examined a potential speed-accuracy trade-off by substituting reaction time with accuracy in both models. The fit of the calculated models was assessed and considered reasonable at χ^2^/df ≤ 3, CFI near 0.95, and RMSEA < 0.08. RMSEA values ranging from 0.08 to 0.10 represented a marginal fit [66, 67].

Post hoc analyses were conducted using linear regression analysis to explore whether factors such as sociodemographic background or baseline scores predicted absolute participation rates. The hypothesis that regression coefficients do not differ from zero was tested and rejected at a significance level of *p* < 0.05.

## Results

### Sample

The study involved 142 participants originating from 10 countries, predominantly Afghanistan (53.5%, *n* = 76), Somalia (15.5%, *n* = 22), the Democratic Republic of the Congo (12.0%, *n* = 17), and Syria (8.5%, *n* = 12). At T1, 30.3% (*n* = 43) of the participants experienced moderate pain, whereas 6.3% (*n* = 9) reported severe pain. Sample characteristics at T1 are presented in Table 2.

**Table 2:** Participant’s sociodemographic background and PTSD symptom severity at T1

	Overall (*n* = 142)	Dropouts (*n* = 44)	WLCG completers (*n* = 49)	IG completers (*n* = 49)
*Sex*				
Women	75 (52.8)	18 (40.9)	28 (57.1)	29 (59.2)
Men	67 (47.2)	26 (59.1)	21 (42.9)	20 (40.8)
*Age (in years)*	29.2 [27.7;30.8]	27.7 [25.5;30.0]	31.9 [29.0;34.9]	27.8 [25.1;30.5]
*Origin*				
South Asia	77 (57.0)	12 (30.8)	29 (60.4)	36 (75.0)
West Asia	18 (13.3)	8 (20.5)	4 (8.3)	6 (12.5)
East Africa	22 (16.4)	11 (28.2)	7 (14.6)	4 (8.3)
Central Africa	18 (13.3)	8 (20.5)	8 (16.7)	2 (4.2)
*Education*				
Uncompleted primary	36 (26.5)	12 (30.0)	13 (27.6)	11 (22.4)
Primary	51 (37.5)	10 (25.0)	17 (36.2)	24 (49.0)
High school and above	49 (36.0)	18 (45.0)	17 (36.2)	14 (28.6)
*Time in camp (in months)*	14.9 [13.2;16.7]	15.5 [12.5;18.5]	15.5 [11.9;19.1]	13.9 [11.3;16.6]
*PTSD (IES-R)*	35.4 [31.7;39.1]	26.4 [20.6;32.1]	33.4 [27.0;39.8]	45.8 [39.6;51.9]

Data are presented as mean [95% CI] and frequency (%)

*IES-R*, Impact of Event Scale-Revised; *IG*, intervention group; *PTSD*, post-traumatic stress disorder; *T1*, baseline; *WLCG*, waitlist control group

**Fig. 1:**
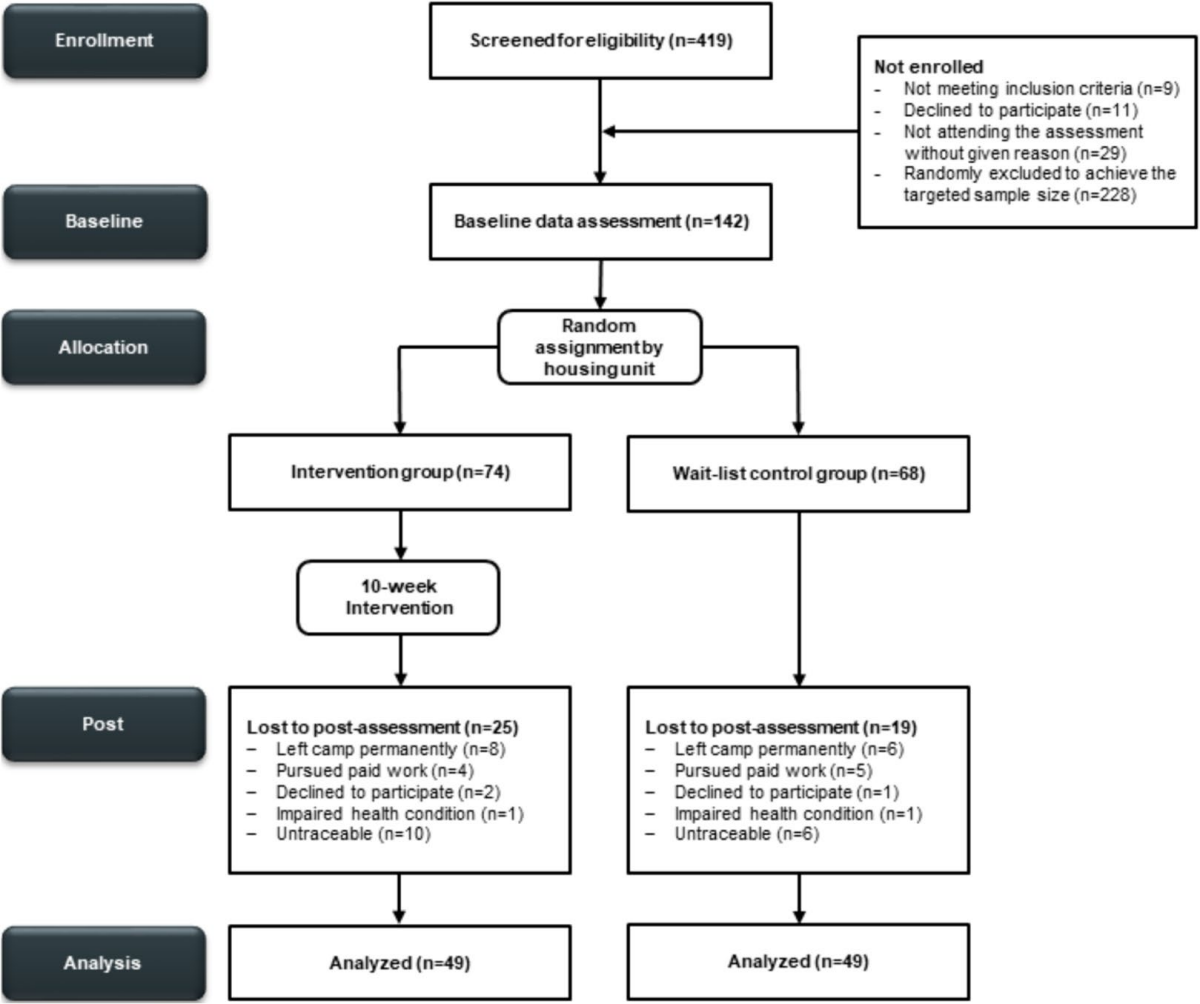
CONSORT flow diagram of the study

In total, 69.0% (*n* = 98) of the participants completed T2, 47.9% (*n* = 68) completed T3, and 35.3% (*n* = 24) completed T4, with T4 including only the WLCG. The high dropout rate can be attributed to the simultaneous start of the intervention with the lifting of COVID-19 measures. Due to the lockdown, asylum applications were put on hold, leading to many individuals receiving their decisions at the same time and subsequently leaving the camp. Dropout at T2 occurred mainly because participants were no longer in the camp for unknown reasons (11.3%, *n* = 16) or had left after completing their asylum procedures (9.9%, *n* = 14). Participants from West Asia, Central, or East Africa, χ^2^(3) = 15.65, *p* = 0.001, with lower pain levels, *t*(70.3) =  − 2.36, *p* = 0.021, and PTSD symptoms, *t*(98.0) =  − 3.59, *p* < 0.001, were more prone dropping out compared to individuals who completed T2. Participant flow is illustrated in Fig. 1. The targeted 1:1 allocation ratio was not fully met, related to household selection and sex-based stratification.

At T1, 11.2% (*n* = 11) of the cases had missing values in the Oddball paradigm and Flanker task, and 9.2% (*n* = 9) at T2, mainly attributed to field measurement difficulties. For pain, 9.2% (*n* = 9) missing values were recorded at T1, with no missing data at T2. We noted 20.4% (*n* = 20) cases of uni- and multivariate missing CRF values at T1 and T2, respectively, due to acute injuries, knee issues, or general discomfort. Little’s missing completely at random test was significant, χ^2^(125) = 152.37, *p* = 0.048, indicating varied probabilities of missing data across cases. Independent samples *t*-tests showed no significant differences in T1 values between groups with complete and incomplete data in the respective outcome at T2 (*p* > 0.160). However, participants with one or more missing values were predominantly female, χ^2^(1) = 20.61, *p* < 0.001, performed slower reaction times in the Oddball paradigm, *t*(72.6) = 2.18, *p* = 0.032, and had lower CRF values, *t*(51.2) =  − 2.52, *p* = 0.015, at T2. As most of the missing data belonged to CRF, post hoc analysis revealed that older participants, *t*(46.1) = 2.17, *p* = 0.035, tended to have incomplete CRF data. Since age was in zero-order correlations positively correlated with reaction time, *r*(87) = 0.47, *p* < 0.001, and negatively with CRF, *r*(76) =  − 0.40, *p* < 0.001, we adjusted our models to control for sex and age, treating the data as missing at random.

Upon outlier analysis, we removed 1 univariate outlier of 91.9 ml/kg/min for CRF (> 3 SDs above the mean). Additionally, multivariate outliers with *p* < 0.001 for Mahalanobis distance were excluded for 3 cases in the Oddball paradigm and 6 cases in the Flanker task.

### Intervention

In our study, 40.8% (*n* = 20) of the participants met the recommended two weekly training sessions, while 32.7% (*n* = 16) participated once per week. Of all sociodemographic factors and baseline scores, only CRF was linked to an increased participation rate, *ß* = 0.41, *p* = 0.007. No serious adverse events were associated with the intervention. However, some non-serious incidents occurred during the activities. One participant experienced heart rate spikes of over 200 bpm during brisk walking and was excluded from the intervention on medical advice. Additionally, 10.2% (*n* = 5) of the participants encountered issues like bruised chest, sprained finger, and shoulder or knee pain, leading to temporary discontinuation of participation for 4 to 30 days. A few participants reported dizziness during and muscle soreness after activities.

### Intervention effects

**Fig. 2:**
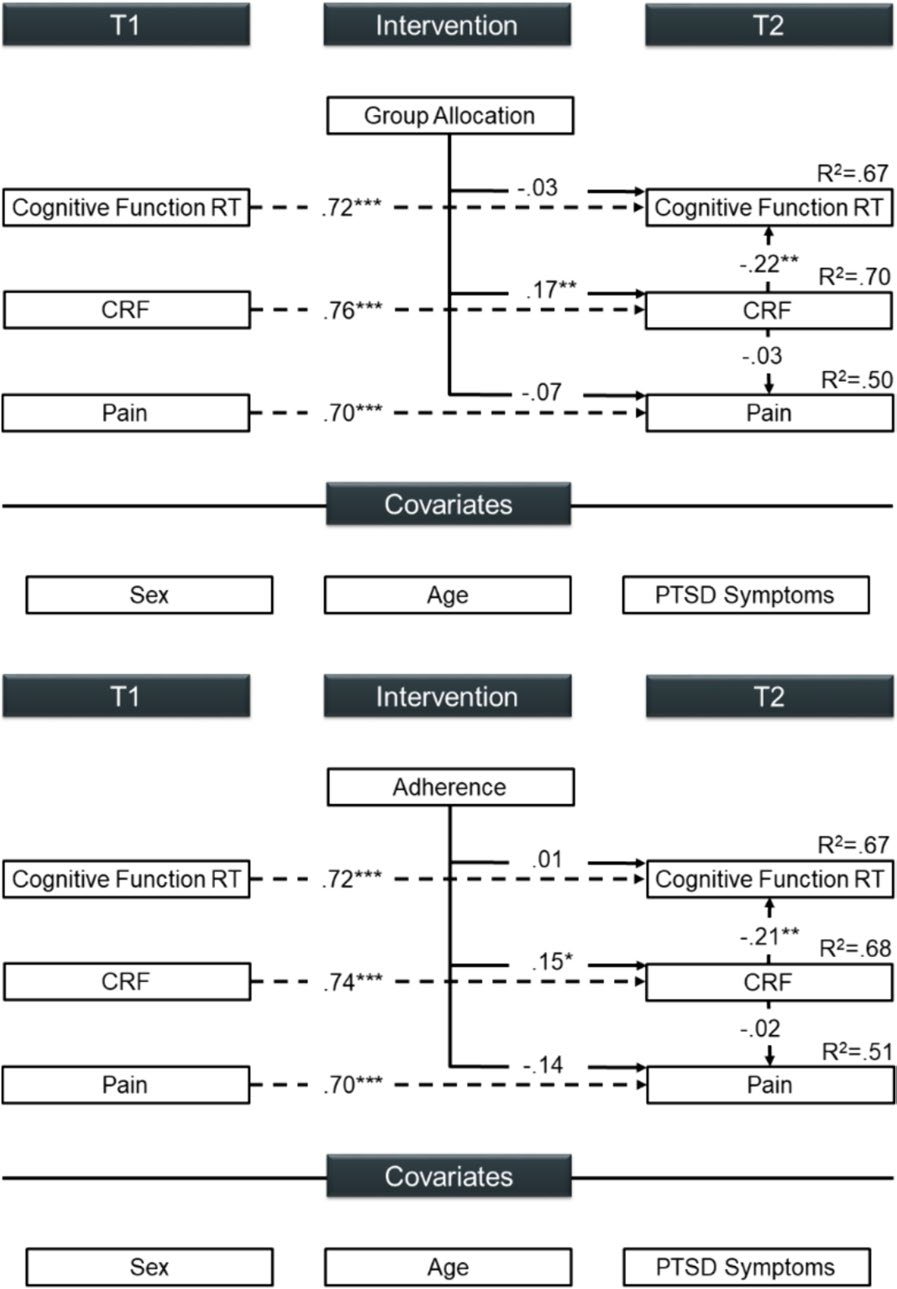
Model examining the effect of group allocation (model 1), adherence (model 2), and CRF on cognitive function and pain. Outcomes were controlled for sex, age, and PTSD symptom severity. Model fit indices were χ^2^/df = 1.845, CFI = .928, and RMSEA = .093 for model 1 and χ^2^/df = 1.500, CFI = .956, and RMSEA = .072 for model 2. Path coefficients are displayed as standardized betas. *p < .05, **p < .01, ***p < .001. Abbreviations: CRF, cardiorespiratory fitness; PTSD, post-traumatic stress disorder; RT, reaction time; T1, baseline; T2, post-intervention

Table 3 outlines the descriptive statistics of the outcomes at T1 and T2. Using standardized path coefficients, Fig. 2 displays the effects on reaction time in cognitive tasks and pain. We found no evidence of a direct intervention effect on reaction time or pain (*p* ≥ 0.332), nor did we observe a trade-off by task accuracy at T2, *ß* =  − 0.11, *p* = 0.197. The IG showed a significant increase in CRF at T2, *ß* = 0.17, *p* = 0.010, with adherence linked to gains in CRF, *ß* = 0.15, *p* = 0.027. Enhanced CRF was associated with faster reaction time in cognitive tasks, *ß* =  − 0.22, *p* = 0.004, but not to pain, *ß* =  − 0.03, *p* = 0.663. As-treated analysis indicated that adherence was neither associated with reaction time nor accuracy in cognitive tasks at T2 (*p* ≥ 0.876). Noteworthy, the data indicated some evidence for pain, implying that regular participation was linked to reduced pain severity at T2, *ß* =  − 0.14, *p* = 0.065. Among the covariates at T1, being female was linked to extended reaction times, *ß* = 0.42, *p* < 0.001, and lower CRF, *ß* =  − 0.56, *p* < 0.001. Age was associated with longer reaction times, *ß* = 0.40, *p* < 0.001, higher levels of pain, *ß* = 0.22, *p* = 0.008, and decreased CRF, *ß* =  − 0.29, *p* < 0.001. While PTSD symptom severity was not linked to reaction times, *ß* = 0.08, *p* = 0.398, it was related to elevated pain levels, *ß* = 0.53, *p* < 0.001. There was some evidence that the subscore for intrusion was associated with longer reaction times, *ß* = 0.16, *p* = 0.064, whereas the subscore for hyperarousal was not, *ß* = 0.03, *p* = 0.695. However, both subscales were associated with pain severity, *ß* = 0.48, *p* < 0.001, and *ß* = 0.57, *p* < 0.001, respectively.

**Table 3:** Outcome measures separated by group allocation and adherence at T1 and T2

	*n*	T1		*n*	T2	
		Control (*n* = 49)	Intervention (*n* = 49)		Control (*n* = 49)	Intervention (*n* = 49)
*Oddball paradigm reaction time (in ms)*	84	510.2 [467.8;552.6]	448.5 [412.6;484.4]	86	467.4 [433.6;501.2]	427.5 [396.9;458.1]
*Oddball paradigm accuracy (in %)*	84	80.4 [74.7;86.0]	78.5 [72.4;84.6]	86	86.2 [82.1;90.3]	82.5 [77.6;87.5]
*Flanker task reaction time (in ms)*	81	544.8 [493.3;596.3]	456.2 [406.6;505.8]	83	494.9 [452.6;537.2]	435.8 [391.2;480.5]
*Flanker task accuracy (in %)*	81	72.9 [66.6;79.2]	78.0 [71.5;84.5]	83	78.2 [73.1;83.2]	77.6 [70.8;84.3]
*Pain (VAS)*	90	28.1 [21.9;34.3]	32.7 [26.0;39.5]	98	29.7 [23.4;36.1]	30.2 [23.9;36.6]
*CRF (in ml/kg/min)*	78	32.6 [29.2;36.1]	32.1 [28.6;35.7]	78	32.0 [28.6;35.4]	37.0 [33.3;40.7]
	*n*	T1		*n*	T2	
		Comparison (*n* = 62)	Treated (*n* = 36)		Comparison (*n* = 62)	Treated (*n* = 36)
*Oddball paradigm reaction time (in ms)*	84	503.1 [466.2;540.0]	440.7 [399.0;482.4]	86	461.2 [432.4;490.0]	425.4 [387.5;463.4]
*Oddball paradigm accuracy (in %)*	84	79.6 [74.3;84.8]	79.3 [72.5;86.0]	86	85.0 [80.7;89.3]	83.4 [78.8;88.0]
*Flanker task reaction time (in ms)*	81	540.8 [494.0;587.5]	436.5 [383.7;489.3]	83	491.7 [451.1;532.2]	424.1 [378.9;469.4]
*Flanker task accuracy (in %)*	81	72.2 [66.2;78.1]	80.7 [74.1;87.3]	83	76.4 [71.2;81.6]	80.4 [73.5;87.3]
*Pain (VAS)*	90	30.5 [24.6;36.5]	30.3 [23.0;37.6]	98	32.2 [26.2;38.2]	26.3 [19.9;32.6]
*CRF (in ml/kg/min)*	78	31.5 [28.5;34.4]	33.7 [29.3;38.0]	78	32.2 [29.1;35.3]	38.1 [33.9;42.3]

Data are presented as mean [95% CI] for rare trials of the Oddball paradigm, incongruent trials of the Flanker task, and accuracy of response-correct trials

*CRF*, cardiorespiratory fitness; *T1*, baseline; *T2*, post-intervention; *VAS*, visual analog scale

## Discussion

The findings of this secondary analysis indicate that the intervention did not directly impact cognitive function and pain. In contrast, enhancements in CRF were found to benefit cognitive function.

The absence of observable direct intervention effects in our study may have been attributable to the specific nature of the intervention. Qualitative and quantitative aspects of exercise and sport activities, namely their type and dosage, have been identified as potential moderators influencing effects on cognitive function. Activities requiring coordination, such as dancing, or martial arts have shown greater effects on executive function compared to resistance or endurance exercise [25]. Those effects are thought to stem from the cognitive demands of specific types of exercise [26]. Additionally, existing research suggested that cognitive function improvements are more pronounced with an increased overall exercise dose [68]. Given that our intervention featured a range of activities with varying cognitive demands and was of short duration, it might not have met the optimal conditions for cognitive enhancement suggested by current studies.

However, our findings indicated that enhancements in CRF corresponded to improved cognitive function. Activities should be conducted at a minimum of moderate intensity, as CRF was reported to be more responsive to intensity rather than duration or frequency [69]. Additionally, our preliminary analysis (Additional file 1: Table S2) showed that the impact of exercise on cognitive function is more general than selective, indicated by only small differences in effect sizes between sustained attention and inhibitory control. This aligns with a recent review [26], which concluded that exercise promotes structural brain changes with improved CRF being associated with increased cerebral blood flow, neuroplasticity, and gray or white matter volume, which, in turn, generally enhances cognitive function.

Our data indicated that the intervention did not reduce pain severity. Although an overview of Cochrane reviews suggested potential benefits of exercise interventions for pain relief, the evidence remained inconsistent and of low quality [16]. The lack of statistical significance in our study’s results might be attributed to the low baseline pain severity observed in our sample, with 63.4% reporting mild and 30.3% reporting moderate pain severity. Therefore, demonstrating a significant change could be more challenging compared to studies with clinical samples reporting more severe pain.

Pain could occur secondary to conditions like mental distress, poor sleep, or physical impairment [16]. Among forcibly displaced individuals in Greece, pain might be exacerbated through psychological stress due to traumatic experiences or post-migration living difficulties [15]. Transcultural psychiatric approaches also highlighted the role of sociocultural factors in understanding somatic symptoms across individuals with different origins. Cultural interpretations and the fear of stigmatization in some West Asian communities could blur the distinction between somatic experience and psychological symptoms. Consequently, pain frequently emerged as a prominent complaint among individuals experiencing mental distress [17]. Our findings indicated a similar connection between PTSD symptoms and pain. This overlap might explain the absence of significant differences in pain outcomes between our intervention and control groups.

Currently, high-quality evidence is insufficient to recommend specific types of exercises or dosages for pain relief [16]. Particularly, only two studies have explored the effects of exercise on pain in forcibly displaced populations combined with cognitive behavioral therapy [28, 29]. Neither study reported a significant reduction in pain, attributing this outcome to low adherence rates. We found in our study a potential link between adherence and reduced pain severity, supporting the notion that the benefits of exercise and sport interventions on pain may be dose-dependent [70].

Conversely, we did not observe a link between improvements in CRF and pain reduction in our sample. This contrasted with a cross-sectional analysis where CRF was associated with lower pain severity [27] but aligned with another study where aerobic fitness training did not mitigate pain in individuals with fibromyalgia [71]. Given that 36.9% in our study experienced moderate or severe pain and that strenuous exercise can exacerbate pain through hyperalgesic effects [70], individuals with pain might have participated at low intensity levels. The absence of a direct relationship between CRF enhancement and pain reduction could stem accordingly from the extended period needed for the exercise-induced benefits to emerge, particularly considering the limitations on exercise intensity and progression imposed by pain [16]. Therefore, interventions for individuals experiencing pain may require longer durations to achieve significant benefits.

Additionally, studies emphasized a complex interplay between cognitive function, pain, psychological distress, and post-migration challenges [6, 13, 16], indicating that simultaneous treatment of these conditions might be essential for effectiveness [17]. Hence, a multidisciplinary approach that addresses the intertwined physical, psychological, and social determinants has been recommended for addressing the health needs of forcibly displaced individuals [6, 36].

Notably, we considered the intervention safe, with most adverse events consisting of temporary increased soreness or muscle pain that diminished after a few weeks into the intervention. This aligned with findings that non-serious adverse events were an increased risk in exercise and sport [58]. Yet, the overall health benefits of physical activity have been reported outweighing these risks, even for individuals with long-term health conditions [72].

Our study stands out by addressing an urgent public health challenge [73] and aligning with the WHO’s latest research agenda concerning health, migration, and displacement [23]. Despite navigating the complexity of an increasingly restrictive environment [73], our broad inclusion criteria and the real-world implementation of our intervention have bolstered the external validity of our findings. This study provides preliminary insights into the feasibility and potential benefits of exercise and sport, representing an initial step in building evidence for community-based interventions with individuals living in a refugee camp.

Nevertheless, our findings should be interpreted with caution due to several limitations. We observed a considerable context-related dropout and missing data, though our results were comparable with prior research [26, 70, 71]. The dropout rate of 31.0% surpassed the anticipated 25.0% in our power calculation, accounting for a small effect and thus heightening the risk of false positive outcomes. Recognizing that certain activity types promote stronger cognitive function than others [25], we prioritized participant autonomy over a more controlled intervention. While offering leisure time activities may better reflect naturalistic application, the self-selected nature of the intervention led to variability in both the type and amount of physical activity. Coupled with the relatively short intervention period of 10 weeks, this variability poses challenges in accurately determining the impact of exercise and sport. The pragmatic design of the study also limited systematic evaluations of control conditions like physical activity, session intensities, amount of sleep, and medication usage (sedatives and analgesics). Lastly, our assessment focused on non-specific pain severity over the preceding week, neglecting the potential impact of acute pain from injuries or infections. Additionally, the intervention group’s younger average age and elevated PTSD symptoms may have influenced the outcome.

## Conclusions

Given the global scope of forced displacement, international organizations emphasize the need for effective and scalable interventions to support individuals’ health and enable meaningful participation in the host society. Co-designed exercise and sport activities did not directly impact cognitive function and pain severity among a sociodemographically diverse sample living in a refugee camp in Greece and, therefore, may need to be complemented by additional measures. Our findings integrate well into current research, emphasizing to focus on adherence in interventions, which might help mitigate pain and to train for improvements in CRF, which positively impacts cognitive function.

## Supplementary Information

Additional file 1: Tables S1–S2. Table S1 CONSORT checklist. Table S2 Standardized effects on outcomes at T2.

## Data Availability

The anonymized data used and analyzed in the current study are available on reasonable request from the corresponding author.

## Abbreviations

95% CI: 95% Confidence interval
CFI: Comparative Fit Index
CONSORT: Consolidated Standards of Reporting Trials
CRF: Cardiorespiratory fitness
DSM-5: Diagnostic and Statistical Manual of Mental Disorders (5th edition)
ICD-10: International Classification of Diseases (10th version)
IES-R: Impact of Event Scale-Revised
IG: Intervention group
ISRCTN trial registry: Primary clinical trial registry recognized by the WHO and ICMJE
M: Mean
PTSD: Post-traumatic stress disorder
RMSEA: Root mean square error of approximation
SPSS: Statistical Package for the Social Sciences
T1: Baseline
T2: Post-intervention
UNHCR: United Nations High Commissioner for Refugees
VAS: Visual analog scale
VO_2_max (ml/kg/min): Maximal oxygen uptake
WHO: World Health Organization
WLCG: Waitlist control group
