# Supplementary material for: The impact of an exercise and sport intervention on cognitive function and pain among forcibly displaced individuals at risk for PTSD: a secondary analysis of the SALEEM randomized controlled trial
Source: BMC Med. 2024 Sep 12;22:387. doi: 10.1186/s12916-024-03601-x (PMC11396323; doi:10.1186/s12916-024-03601-x)
Supplement: Supplementary file 1 — Additional file 1: Tables S1–S2. Table S1 CONSORT checklist. Table S2 Standardized effects on outcomes at T2. [file 12916_2024_3601_MOESM1_ESM.docx]

**SUPPLEMENTAL DIGITAL CONTENT**

**Table S1.** CONSORT checklist.

| Section/Topic | Item No | Checklist item | Page No |
| --- | --- | --- | --- |
|  |  | Title and abstract |  |
|  | 1a | Identification as a randomised trial in the title | 1 |
|  | 1b | Structured summary of trial design, methods, results, and conclusions (for specific guidance see CONSORT for abstracts) | 1 |
|  |  | Introduction |  |
| Background and objectives | 2a | Scientific background and explanation of rationale | 2 |
|  | 2b | Specific objectives or hypotheses | 3 |
|  |  | Methods |  |
| Trial design | 3a | Description of trial design (such as parallel, factorial) including allocation ratio | 3 |
|  | 3b | Important changes to methods after trial commencement (such as eligibility criteria), with reasons | NA |
| Participants | 4a | Eligibility criteria for participants | 3 |
|  | 4b | Settings and locations where the data were collected | 3 |
| Interventions | 5 | The interventions for each group with sufficient details to allow replication, including how and when they were actually administered | 4 |
| Outcomes | 6a | Completely defined pre-specified primary and secondary outcome measures, including how and when they were assessed | 5 |
|  | 6b | Any changes to trial outcomes after the trial commenced, with reasons | NA |
| Sample size | 7a | How sample size was determined | 3 |
|  | 7b | When applicable, explanation of any interim analyses and stopping guidelines | NA |
|  |  | Randomisation: |  |
| Sequence generation | 8a | Method used to generate the random allocation sequence | 3 |
|  | 8b | Type of randomisation; details of any restriction (such as blocking and block size) | 3 |
| Allocation concealment mechanism | 9 | Mechanism used to implement the random allocation sequence (such as sequentially numbered containers), describing any steps taken to conceal the sequence until interventions were assigned | 3 |
| Implementation | 10 | Who generated the random allocation sequence, who enrolled participants, and who assigned participants to interventions | 3 |
| Blinding | 11a | If done, who was blinded after assignment to interventions (for example, participants, care providers, those assessing outcomes) and how | 5-6 |
|  | 11b | If relevant, description of the similarity of interventions | NA |
| Statistical methods | 12a | Statistical methods used to compare groups for primary and secondary outcomes | 6 |
|  | 12b | Methods for additional analyses, such as subgroup analyses and adjusted analyses | 6 |
|  |  | Results |  |
| Participant flow | 13a | For each group, the numbers of participants who were randomly assigned, received intended treatment, and were analysed for the primary outcome | 8 |
|  | 13b | For each group, losses and exclusions after randomisation, together with reasons | 8 |
| Recruitment | 14a | Dates defining the periods of recruitment and follow-up | 3-4 |
|  | 14b | Why the trial ended or was stopped | NA |
| Baseline data | 15 | A table showing baseline demographic and clinical characteristics for each group | 7 & 9 |
| Numbers analysed | 16 | For each group, number of participants (denominator) included in each analysis and whether the analysis was by original assigned groups | 9 |
| Outcomes and estimation | 17a | For each primary and secondary outcome, results for each group, and the estimated effect size and its precision (such as 95% confidence interval) | 8-10 |
|  | 17b | For binary outcomes, presentation of both absolute and relative effect sizes is recommended | NA |
| Ancillary analyses | 18 | Results of any other analyses performed, including subgroup analyses and adjusted analyses, distinguishing pre-specified from exploratory | 7 |
| Harms | 19 | All important harms or unintended effects in each group (for specific guidance see CONSORT for harms) | 8 |
|  |  | Discussion |  |
| Limitations | 20 | Trial limitations, addressing sources of potential bias, imprecision, and, if relevant, multiplicity of analyses | 10-11 |
| Generalisability | 21 | Generalisability (external validity, applicability) of the trial findings | 10 |
| Interpretation | 22 | Interpretation consistent with results, balancing benefits and harms, and considering other relevant evidence | 9-10 |
|  |  | Other information |  |
| Registration | 23 | Registration number and name of trial registry | 1 |
| Protocol | 24 | Where the full trial protocol can be accessed, if available | 3 |
| Funding | 25 | Sources of funding and other support (such as supply of drugs), role of funders | 11 |

**Table S2.** Standardized effects on outcomes at T2.

|  | R^2^ | Group | CRF T2 | Modelfit | | |
| --- | --- | --- | --- | --- | --- | --- |
|  |  |  |  | χ2/df | CFI | RMSEA |
| *Oddball paradigm (RT)* | .44 | -.01 (.876) | -.22 (.016) | 1.824 | .920 | .092 |
| *Oddball paradigm (ACC)* | .21 | -.09 (.349) | -.06 (.564) | 1.845 | .896 | .093 |
| *Flanker task (RT)* | .55 | -.01 (.890) | -.22 (.009) | 2.109 | .902 | .107 |
| *Flanker task (ACC)* | .58 | -.13 (.086) | .03 (.666) | 1.711 | .926 | .086 |
| *Cognitive Function (RT)* | .67 | -.03 (.666) | -.22 (.004) | 1.845 | .928 | .093 |
| *Cognitive Function (ACC)* | .46 | -.11 (.197) | .04 (.642) | 1.703 | .920 | .085 |
| Note: Effects are presented as standardized path coefficients (p-value). Abbreviations: ACC = Accuracy; CRF = Cardiorespiratory fitness; RT = Reaction time; T2 = Post-intervention. | | | | | | |
